# Cleavage of an RNA Model Compound by an Arylmercury Complex

**DOI:** 10.1002/cbic.202000799

**Published:** 2021-02-24

**Authors:** Lange Yakubu Saleh, Mikko Ora, Tuomas Lönnberg

**Affiliations:** ^1^ Department of Chemistry University of Turku Vatselankatu 2 20014 Turku Finland

**Keywords:** catalysis, cleavage, mercury, organometallic catalysts, phosphodiester, RNA

## Abstract

A water‐soluble arylmercury complex has been synthesized, and its ability to catalyze the cleavage of the phosphodiester linkage of the RNA model compound adenylyl‐3′,5′‐(2′,3′‐*O*‐methyleneadenosine) has been assessed over a pH range of 3–8.5 and a catalyst concentration range of 0–7 mM. In the presence of 1 mM catalyst, the observed pH–rate profile featured a new pH‐independent region between pH 6 and 7, the catalyzed reaction being as much as eight times faster than the background reaction. At pH 7, the acceleration increased linearly from three‐ to 17‐fold upon increasing the catalyst concentration from 1 to 7 mM. The linear dependence indicates a relatively low affinity of the catalyst for the substrate and, hence, the potential for considerable improvement on tethering to an appropriate targeting group, such as an oligonucleotide.

## Graphical abstract

**Soluble, but not so speedy**: The potential of an water‐soluble arylmercury compound to cleave RNA has been explored. The somewhat low catalytic activity compared to free Hg^II^ was attributed to the relatively low affinity of the arylmercury catalyst for the RNA model compound. This suggests that improved derivatives could be obtained by covalent tethering to an appropriate targeting group.

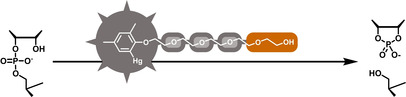

The design of antisense oligonucleotides typically involves a trade‐off between cellular uptake, nuclease stability and the ability to activate RNase H. For example, extensively modified antisense oligonucleotides may be highly resistant towards degradation by cellular nucleases but at the same time their heteroduplexes with complementary RNA are no longer substrates for RNase H. Antisense oligonucleotides that can catalyze the cleavage of a complementary RNA independently of RNase H would offer a solution to this problem and thus largely remove limitations regarding the extent of applicable modifications.

Despite recent advances in development of metal‐free RNA‐cleaving agents,[^1^, ^2^, ^3^] the most efficient artificial ribonucleases employ coordination complexes of catalytic metal ions such as Cu^II^ and Zn^II^.[^4^, ^5^, ^6^, ^7^, ^8^, ^9^, ^10^, ^11^, ^12^, ^13^, ^14^] Unfortunately, these complexes dissociate easily under highly diluted and metal‐deficient conditions such as those of the intracellular medium. For this reason, there is demand for artificial ribonucleases combining the stability of organic molecules with the efficiency of metal catalysts, especially for therapeutic applications. Organometallic complexes could, in principle, meet both of these requirements but, to the best of our knowledge, have not been systematically studied as RNA‐cleaving agents.

We have recently tested the catalytic effect of a free metal ion, Hg^II^, on the cleavage of RNA with a simple dinucleoside monophosphate model compound as well as its phosphoromonothioate analogs.^[15]^ The solubility of Hg^II^ limited the studies to a relatively narrow pH range excluding physiological conditions. At pH 5.0, a more than 100‐fold acceleration of the cleavage of an RNA phosphodiester linkage could be attained, comparable to many other divalent metal ions.^[16]^ Encouraged by this result, we next set out to explore the potential of an arylmercury compound to catalyze the cleavage of the phosphodiester linkage of the same dinucleoside monophosphate model compound. Arylmercury compounds are hydrolytically stable and readily accessible through treatment of the parent arene with Hg^II^ salts, making them attractive as a first generation of organometallic RNA‐cleaving agents. A hydrophilic penta(ethylene glycol) tail was incorporated to improve the aqueous solubility of the catalyst. Oligo‐ and poly(ethylene glycol) conjugation is perhaps the most popular and best‐documented strategy for this purpose and has been used to solubilize such diverse species as small molecular drugs, peptides, proteins, oligonucleotides and nanoparticles.[^17^, ^18^, ^19^, ^20^] As the well‐known toxicity of organomercury compounds is related to their lipophilic bioaccumulation, this modification would be expected to make the cleaving agent less toxic. The results of these experiments are reported in the present paper and compared with those previously obtained using Hg^II^ as a catalyst.

The organometallic catalyst **1**‐Hg was synthesized as described in Scheme 1. Penta(ethylene glycol) was first tosylated in the presence of silver oxide and sodium iodide in dichloromethane, affording intermediate **2**. The tosylate group was displaced from **2** with 2,4‐dimethylphenol in the presence of potassium carbonate in dimethylformamide. Finally, the intermediate **3** formed was treated with mercuric acetate in CD_3_OD to produce **1**‐Hg.

**Scheme 1:**
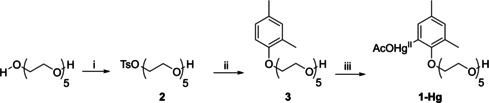
Synthesis of the organomercury catalyst **1**‐Hg. i) Ag_2_O, NaI, TsCl, dry CH_2_Cl_2_; ii) K_2_CO_3_, 2,4‐dimethylphenol, dry DMF; iii) Hg(OAc) _2_, CD_3_OD.

Cleavage of adenylyl‐3′,5′‐(2′,3′‐*O*‐methyleneadenosine)^[15]^ (**4**) was followed as a function of pH (3.0–8.5) in the absence and presence of **1**‐Hg (1 mM) at 90 °C by analyzing the composition of the aliquots withdrawn from the reaction mixture by RP‐HPLC. The improved aqueous solubility of compound **1**‐Hg, featuring a hydrophilic penta(ethylene glycol) tail, allowed considerable expansion of the pH range compared to the previous study with Hg^II^, as evidenced by both visual inspection of the clarity of the reaction solutions as well as the fact that catalysis by **1**‐Hg prevailed under conditions where catalysis by Hg^II^ could not be detected. Irrespective of the presence of **1**‐Hg, the starting material **4** was partly converted to its 2′,5′‐isomer (**5**) and the isomeric mixture was cleaved to 2′,3′‐*O*‐methyleneadenosine (**6**) and 2′,3′‐cAMP which, in turn, was subsequently hydrolyzed to adenosine via 2′/3′‐AMP (Scheme 2).

**Scheme 2:**
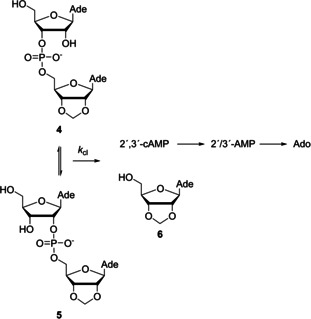
Hydrolysis of ApA derivatives **4** and **5**.

Under acidic (pH<5) and basic (pH>7) conditions, the pH–rate profiles for the cleavage of **4** and **5** were quite similar in the absence and presence of 1 mM **1**‐Hg, as seen from Figure 1. Under neutral and slightly acidic conditions, however, a significant difference was observed. In the absence of **1**‐Hg, the pH–rate profile consisted of a hydronium ion‐catalyzed reaction (first order in [H^+^]) at pH<5, nearly pH‐independent reaction from pH 5 to 6 and a hydroxide ion‐catalyzed reaction (first order in [OH^−^]) at pH>6, in line with previous reports on related systems.[^21^, ^22^] The **1**‐Hg‐catalyzed cleavage, in turn, exhibited an additional pH‐independent plateau at pH 6–7 and a second‐order dependence on [OH^−^] at pH 5–6. At pH 6, cleavage of **4** and **5** was eight times faster in the presence of 1 mM **1**‐Hg. For reference, a 17‐fold rate enhancement by free Hg^II^ has been reported under the same conditions.^[15]^ The rate of mutual isomerization of **4** and **5** was not appreciably affected by the presence of **1**‐Hg.

**Figure 1:**
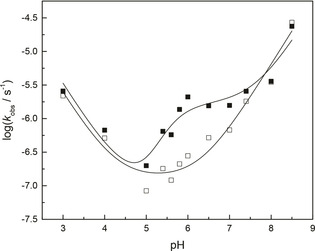
pH–rate profiles for the cleavage (*k*_cl_) of a mixture of adenylyl‐3′,5′‐(2′,3′‐*O*‐methyleneadenosine) (**4**) and its 2′,5′ isomer (**5**) in the absence (□) and presence (▪) of **1**‐Hg; *T=*90 °C, [**1**‐Hg]=1 mM, [buffer]=30 mM; *I*(NaNO_3_)=100 mM.

The observed rate constant for the cleavage of **4** and **5** (*k*_cl_) may be expressed by Equation 1.

(1) kobscl=kHcl[H+]+kH2Ocl+kcatcl[1-Hg]Ka1Ka2[H+]2Ka1[H+]+Ka1Ka2+kOHclKW[H+]

kH2Ocl is the first‐order rate constant for the pH‐independent cleavage, kHcl , kOHcl and kcatcl the second‐order rate constants for the hydronium ion, hydroxide ion and **1**‐Hg‐catalyzed cleavage, respectively, and *K*_W_ the ion product of water under the experimental conditions (6.2×10^−13^ M^2^). The chemical significance of the kinetic acid dissociation constants *K*_a1_ and *K*_a2_ remained obscure but they are probably related to protolytic equilibria at a Hg^II^ aqua ligand and/or an adenine base. The apparent second‐order dependence on [OH^−^] between pH 5 and 6 suggests that the values of these two constants must be very similar and, in fact, they could not be determined independently. With this exception, all parameters were determined by nonlinear least‐squares fitting of the experimental data to Equation (1) and the results are summarized in Table 1.

**Table 1:** Rate and kinetic acid dissociation constants for the partial reactions contributing to the cleavage of **4** and **5**; *T*=90 °C, [**1**‐Hg]=0/1 mM, [buffer]=30 mM, *I*(NaNO_3_)=100 mM.

	No catalyst	1.0 mM **1**‐Hg
kHcl /10^−3^ M^−1^ s^−1^	2.4±0.9	3±1
kH2Ocl /10^−7^ s^−1^	1.3±0.3	1±2
kOHcl /M^−1^ s^−1^	0.10±0.02	0.07±0.03
kcatcl /10^−3^ M^−1^ s^−1^	n.a.	1.6±0.5
*K*_a1_=*K*_a2_/10^−6^ M	n.a.	3±2

Dependence of the rate of cleavage of **4** and **5** on the concentration of the organomercury catalyst **1**‐Hg was studied over a range of 0–7 mM at pH 7.0 (30 mM HEPES buffer) under otherwise the same conditions as described above for determination of the pH–rate profile. At higher concentrations, the reaction solution turned turbid, most likely owing to precipitation of **1**‐Hg. Solubility of **1**‐Hg thus precluded kinetic measurements at higher concentrations. Over the concentration range studied, the reaction was first‐order in [**1**‐Hg] and the rate acceleration ranged from three‐ to 17‐fold [**1**‐Hg] (Figure 2). In contrast to the previous report on the catalysis of the same reaction by Hg^II^,^[15]^ the plot was essentially linear, indicating that the association constant for the reactive complex between **4** (or **5**) and **1**‐Hg is too low to be determined given the limited solubility of **1**‐Hg. In line with this result, the association constant of MeHg^II^ has been found to be more than an order of magnitude lower than that of Hg^II^ with both adenine ring nitrogens as well as with oxygen donors.[^23^, ^24^] An apparent second‐order rate constant for the **1**‐Hg‐catalyzed reaction, kcatcl =(1.7±2)×10^−3^ M^−1^ s^−1^, was obtained as the slope of the linear plot. The value is in excellent agreement with the one obtained by fitting the pH‐dependent data to Equation (1).

**Figure 2:**
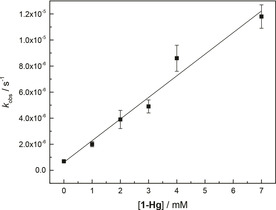
[**1**‐Hg] dependence of the rate of cleavage (*k*_cl_) of **4** and **5**; *T=*90 °C, pH 7.0, [buffer]=30 mM ([HEPES−Na]/[HEPES] 2 : 1), *I*(NaNO_3_)=100 mM. The error bars refer to standard errors of the observed rate constants.

The observation that the mutual isomerization of **4** and **5** is not catalyzed by **1**‐Hg parallels previous reports on catalysis by other metal ions^[22]^ and implies that the cleavage of **4** and **5** is largely attributable to assistance of the departure of the 5′‐linked nucleoside, by either direct coordination of Hg^II^ on the 5′‐oxygen or proton transfer from an aqua ligand. The latter alternative appears more likely as it provides a reasonable mechanistic interpretation for one of the observed kinetic acid dissociation constants, namely deprotonation of a Hg^II^ aqua ligand. Accordingly, a proton would be transferred from the attacking 2′‐oxygen to a Hg^II^ hydroxo ligand in a rapid pre‐equilibrium step and then from a Hg^II^ aqua ligand to the departing 5′‐O concerted with rate‐limiting P−O bond fission (Scheme 3). A similar mechanism has been proposed previously for the cleavage of a dinucleoside‐3′,3′‐phosphodiester model compound having an unprotonated amino group vicinal to the departing 3′‐oxygen.^[25]^ The other kinetic acid dissociation constant is probably related to formation of the reactive complex, presumably through association with the adenine base of the departing nucleoside. The exact nature of this interaction remains obscure but the concomitant loss of a proton suggests that coordination to the exocyclic amino group[^26^, ^27^] might be involved.

**Scheme 3:**
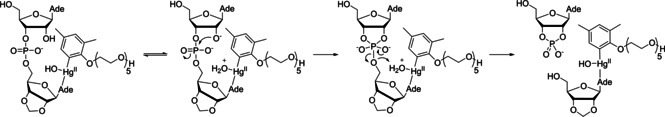
Proposed mechanism for the cleavage of **4** catalyzed by **1**‐Hg.

In summary, the feasibility of organomercury compounds as a new class of RNA‐cleaving agents has been demonstrated. The rate acceleration attained was somewhat lower than that previously reported for Hg^II^ but the difference appears to stem mainly from the lower affinity of the organomercury cleaving agent for the phosphodiester model compound. Tethering to a suitable targeting group, such as a 2′‐*O*‐methyl‐RNA or PNA oligonucleotide, could, hence, greatly improve the efficiency through increased local concentration around the scissile phosphodiester linkage.

## Conflict of interest

The authors declare no conflict of interest.

## Supporting information

As a service to our authors and readers, this journal provides supporting information supplied by the authors. Such materials are peer reviewed and may be re‐organized for online delivery, but are not copy‐edited or typeset. Technical support issues arising from supporting information (other than missing files) should be addressed to the authors.

Supplementary
