# Supplementary material for: Cleavage of an RNA Model Compound by an Arylmercury Complex
Source: Chembiochem. 2021 Feb 24;22(10):1761–4. doi: 10.1002/cbic.202000799 (PMC8247959; doi:10.1002/cbic.202000799)
Supplement: Supplementary file 1 — Supplementary [file CBIC-22-1761-s001.pdf]

ChemBioChem

Supporting Information

Cleavage of an RNA Model Compound by an Arylmercury Complex

Lange Yakubu Saleh, Mikko Ora, and Tuomas Lönnberg*

Contents

General methods ... S2

Kinetic measurements ... S2

An illustrative example of a kinetic measurement ... S3

Figure S1. Linear fitting of $\ln(x(4+5)_t / x(4+5)_0)$ vs. t ... S4

Pentaethylene glycol monotosylate (2) ... S5

14-(2,4-Dimethylphenoxy)-3,6,9,12-tetraoxatetradecan-1-ol (3) ... S5

14-(2,4-Dimethyl-6-mercuryphenoxy)-3,6,9,12-tetraoxatetradecan-1-ol (1-Hg). ... S5

Figure S2. ^{1}H NMR spectra (500 MHz, $CDCl_3$) of compound 2. ... S7

Figure S3. ^{13}C NMR spectra (126 MHz, CDCl3) of compound 2 ... S8

Figure S4. ^{1}H NMR spectra (500 MHz, CD_3OD) of compound 3. ... S9-S10

Figure S5. ^{13}C NMR spectra (126 MHz, CD_3OD) of compound 3. ... S11

Figure S6. ^{1}H NMR spectrum (500 MHz, D_2O) of compound 1-Hg. ... S12

Figure S7. ^{13}C NMR spectra (126 MHz, D_2O) of compound 1-Hg. ... S13-S14

Figure S8. HPLC trace of a product mixture of cleavage and isomerization of 4 and 5 ... S15

General methods

Dichloromethane and *N*,*N*-dimethylformamide were dried over 4 Å molecular sieves. NMR spectra were measured on a Bruker Biospin 500 MHz NMR spectrometer and the chemical shifts (δ, ppm) are quoted relative to the residual solvent peak as an internal standard. High-resolution mass spectra were recorded on a Bruker Daltonics micrOTOF-Q mass spectrometer using electrospray ionization.

Kinetic measurements

The hydrolytic reactions of adenylyl-3´,5´-(2´,3´-*O*-methyleneadenosine) (**4**) were carried out in the absence and presence of the organometallic catalyst **1-Hg** in sealed tubes immersed in a water bath, the temperature of which was kept at 90 °C (± 0.1 °C). The pH of the reaction solutions was adjusted with 0.030 M sodium formate, 2-(*N*-morpholino)ethanesulfonic acid (MES), *N*-(2-hydroxyethyl)piperazine-*N*´-2-ethanesulfonic acid (HEPES) and glycine buffers and the ionic strength (I = 0.10 M) with $NaNO_3$. Before starting to follow the reactions in the presence of **1-Hg**, the pH of the reaction solutions was adjusted exactly to the value measured for the respective reaction solutions in the absence of **1-Hg** by titrating with either 1.00 M NaOH or 0.88 M HNO_3.

125 µL samples were withdrawn from the reaction solutions at appropriate time intervals and cooled to 0 °C to quench the hydrolysis. Composition of the samples was determined by RP-HPLC on a Thermo Scientific Aquasil column (4 × 150 mm, 5 µm) eluting with a linear gradient of acetonitrtile (3—15% over 30 min) in 50 mM aqueous ammonium acetate. The flow rate was 1.0 mL min^{-1} and the detection wavelength 260 nm. The observed retention times (min) were 9.8 (cAMP), 13.7 (adenosine), 19.4 (**5**), 24.4 (**6**) and 26.6 (**4**). The products were identified by spiking with authentic samples as well as by HPLC/MS analysis. A representative chromatogram has been included as Figure S8.

Irrespective of the presence of **1-Hg**, the pH—rate profile for the cleavage of 4 and 5 features a pH-independent minimum flanked by an acid-catalyzed (first-order in [H$^+$]) region under more acidic conditions and a base-catalyzed (first-order in [OH$^-$]) region under more basic conditions. The kinetics of these partial reaction are described by the rate equations (S1), (S2) and (S3), respectively.

$$-\frac{d[\mathbf{4}+\mathbf{5}]}{dt} = k_{cl}^{H2O}[\mathbf{4} + \mathbf{5}] \quad \text{(S1)}$$

$$-\frac{d[\mathbf{4}+\mathbf{5}]}{dt} = k_{cl}^{H}[\mathrm{H}^+][\mathbf{4} + \mathbf{5}] \quad \text{(S2)}$$

$$-\frac{d[\mathbf{4}+\mathbf{5}]}{dt} = k_{cl}^{OH}[\mathrm{OH}^-][\mathbf{4} + \mathbf{5}] = k_{cl}^{OH}\frac{K_W}{[H^+]}[\mathbf{4} + \mathbf{5}] \quad \text{(S3)}$$

k_{cl}^{H2O} is the first-order rate constant for the pH-independent reaction and k_{cl}^{H} and k_{cl}^{OH} the second-order rate constants for the hydronium and hydroxide ion catalyzed reactions. K_W is the ion product of water under

the experimental conditions (6.2×10^{-13} M^2) and [**4**+**5**] the total concentration of compounds **4** and **5**. In the presence of **1-Hg**, an additional plateau at pH 6—7 and a second-order dependence on [OH^-] at pH 5—6 are observed. The second-order dependence on [OH^-] indicates the loss of two protons on going from the dominant ionic form of the complex formed by **4** or **5** with **1-Hg** to the transition state of the **1-Hg** catalyzed reaction. The concentration of this doubly deprotonated species is given by equation (S4) and the kinetics of the **1-Hg** catalyzed partial reaction by equation (S5).

$$[reactive\ complex] = \frac{K_{a1}K_{a2}}{[\mathrm{H}^+]^2 + K_{a1}[\mathrm{H}^+] + K_{a1}K_{a2}}[\mathbf{1-Hg}][\mathbf{4}+\mathbf{5}] \quad \text{(S4)}$$

$$-\frac{d[\mathbf{4+5}]}{dt} = k_{cl}^{cat}\frac{K_{a1}K_{a2}}{[\mathrm{H}^+]^2 + K_{a1}[\mathrm{H}^+] + K_{a1}K_{a2}}[\mathbf{1-Hg}][\mathbf{4}+\mathbf{5}] \quad \text{(S5)}$$

K_{a1} and K_{a2} are acid dissociation constants related to the two deprotonation events to give the reactive complex, k_{cl}^{cat} is the second-order rate constant for the **1-Hg** catalyzed reaction and [1-Hg] the total concentration of **1-Hg**. Finally, the observed rate constant at any pH is given by equation (S6) as the sum of the pseudo first-order rate constants (including the concentration of hydronium ion, hydroxide ion and 1-Hg in case of the respective catalyzed reactions) of the various partial reactions.

$$k_{cl}^{obs} = k_{cl}^{H}[\mathrm{H}^+] + k_{cl}^{H2O} + \frac{k_{cl}^{cat}[\mathbf{1-Hg}]K_{a1}K_{a2}}{[\mathrm{H}^+]^2 + K_{a1}[\mathrm{H}^+] + K_{a1}K_{a2}} + \frac{k_{cl}^{OH}K_W}{[\mathrm{H}^+]} \quad \text{(S6)}$$

Non-linear least-squares fitting of the experimental data to equation (S6) was carried out using Origin 2016 software. The initial guesses for the fitted parameters were $k_{cl}^{H} = 1 \times 10^{-3}$, $k_{cl}^{H2O} = 1 \times 10^{-7}$, $k_{cl}^{OH} = 0.1$, $k_{cl}^{cat} = 1 \times 10^{-6}$ and $K_{a1} = K_{a2} = 3 \times 10^{-6}$. K_W was locked to 6.2×10^{-13}.

An illustrative example of a kinetic measurement

Sodium nitrate (13.1 mg), 200 µL of HEPES buffer (200 mM HEPES sodium salt and 100 mM HEPES), 40 µL of aqueous solution of **4** (30 mM) and 200 µL of aqueous solution of **1-Hg** (30 mM) were added into a 2 mL volumetric flask. The flask was filled to the mark with Milli-Q water and the pH of the resulting solution was adjusted with 1.00 M NaOH to exactly the value measured in the absence of **1-Hg**. The reaction solution was then placed in a sealed tube immersed in a thermostated oil/water bath (90.0 °C ± 0.1 °C). The samples (125 µL) were withdrawn from the reaction solution at appropriate time intervals (Table S1) and were cooled to 0 °C to quench the reactions. Composition of the samples was analyzed by RP-HPLC and the observed peak areas were converted to mole fractions by dividing with the combined peak area of all reaction components (Table S1). Molar absorptivity of the dimeric compounds **4** and **5** was assumed to be twice as high as that of the monomeric compounds 2´,3´-cAMP and **6**. Pseudo first-order rate constants for the decomposition of **4** and its 2´,5´-isomer **5** were obtained by applying the integrated first-order rate equation to the time-dependent combined concentration of these compounds. Accordingly,

ln(x(**4**+**5**)$_t$ / x(**4**+**5**)$_0$) was plotted as a function of t and the rate constant was obtained as the opposite number of the slope of a straight line fitted to the experimental data points (Figure S1).

Table S1. Sampling times and compositions of samples withdrawn from the reaction mixture of compound **4**; T = 90 °C; pH = 7.0; [**1-Hg**] = 3.0 mM; [buffer] = 30 mM ([HEPES-Na]:[HEPES] = 2:1); I($NaNO_3$) = 100 mM.

t / s	x(2´,3´-AMP)	x(**4**+**5**)	x(**6**)
0	0.0038	0.9874	0.0088
2940	0.0228	0.9471	0.0300
6600	0.0200	0.9469	0.0332
10020	0.0314	0.9284	0.0402
18480	0.0457	0.8898	0.0646
27720	0.0591	0.8655	0.0755

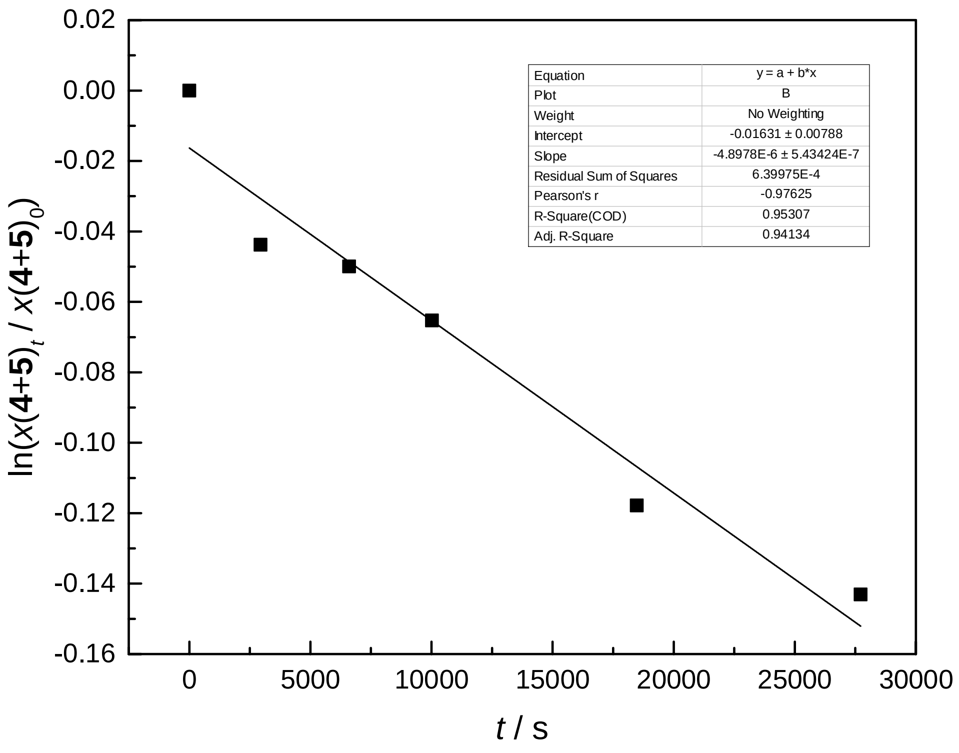

Figure S1. Linear fitting of ln(x(**4**+**5**)$_t$ / x(**4**+**5**)$_0$) vs. t; T = 90 °C; pH = 7.0; [**1-Hg**] = 3.0 mM; [buffer] = 30 mM ([HEPES-Na]:[HEPES] = 2:1); I($NaNO_3$) = 100 mM. Pseudo first-order rate constant was obtained as the opposite number of the slope.

Pentaethylene glycol monotosylate (2)

Pentaethylene glycol (1.91 g, 8.00 mmol) was dissolved in dry dichloromethane (50 mL). The solution was cooled on an ice—water bath and silver oxide (2.78 g, 12 mmol) and sodium iodide (1.27 g, 8.80 mmol) were added. Finally, 4-toluenesulfonyl chloride (1.6 g, 8.40 mmol) was added in small portions over 15 min. The ice—water bath was removed and the reaction mixture was stirred for 10 min at room temperature. The mixture was filtrated and the filtrate washed with a 10% aqueous solution of sodium bicarbonate (50 ml). The organic layer was dried with sodium sulfate, filtrated and evaporated to dryness. Compound **2** was obtained as a colorless liquid in 99% yield (3.12 g). ^{1}H NMR (500 MHz, $CDCl_3$) *δ*: 7.82 (d, *J* = 8.3 Hz, 2H, Ar-H), 7.36 (d, *J* = 8.1 Hz, 2H, Ar-H), 4.19 (t, *J* = 5.0 Hz, 2H, CH_2OTs), 3.75 – 3.60 (m, 18H, 9 × CH_2), 2.47 (s, 3H, Ar-CH_3). ^{13}C NMR (126 MHz, $CDCl_3$) *δ*: 144.8 (Ar-C1), 133.1 (Ar-C4), 129.8, 128.0 (Ar-C3, Ar-C5, Ar-C2 & Ar-C6), 72.5 (CH_2), 70.8 (CH_2), 70.6 (CH_2), 70.6 (CH_2), 70.6 (CH_2), 70.5 (CH_2), 70.4 (CH_2), 69.2 (CH_2), 68.7 (CH_2), 61.8 (CH_2OH), 21.6 (Ar-CH_3). HRMS (ESI) *m/z*: $[M + Na]^+$ calcd for $C_{17}H_{28}O_8SNa^+$ 415.1403, found 415.1391.

14-(2,4-Dimethylphenoxy)-3,6,9,12-tetraoxatetradecan-1-ol (3)

2,4-Dimethylphenol (305 mg, 2.50 mmol) and potassium carbonate (1.73 g, 12.5 mmol) in dry *N*,*N*-dimethylformamide (5.0 mL) was heated to 80 °C and the mixture was stirred for 5 min. A solution of compound **2** (981 mg, 2.5 mmol) in dry *N*,*N*-dimethylformamide (1.0 mL) was added dropwise over 10 min and the reaction mixture was refluxed overnight and then allowed to cool down to room temperature. Cold water was added and the crude product was extracted with dichloromethane, dried with sodium sulfate, filtered and concentrated at reduced pressure. The residue was purified on a silica gel column (flash chromatography) eluting with a mixture of EtOAc and MeOH (9:1, *v/v*). Compound **3** was obtained as a light brown liquid in 55% yield (187 mg). ^{1}H NMR (500 MHz, CD_3OD) *δ*: 6.93-6.91 (m, 2H, Ar-H3 & Ar-H5), 6.76 (d, *J* = 8.0 Hz, 1H, Ar-H6), 4.07 (dd, *J* = 4.5 Hz, 6.5 Hz, 2H, $ArOCH_2$), 3.83 (dd, *J* = 3.5 Hz, 5.0 Hz, 2H, $ArOCH_2C\underline{H}_2O$), 3.72 (m, 2H, $C\underline{H}_2OH$), 3.67 – 3.61 (m, 12H, 6 × CH_2), 3.55 (m, 2H, CH_2), 2.23 (s, 3H, Ar-CH_3), 2.18 (s, 3H, Ar-CH_3). ^{13}C NMR (126 MHz, CD_3OD) *δ*: 154.9 (Ar-C1), 131.0, 126.7 (Ar-C3 & Ar-C5), 129.4, 126.2 (Ar-C2 & Ar-C4), 111.2 (Ar-C6), 72.3 (CH_2), 70.5 (CH_2), 70.2 (CH_2), 70.2 (CH_2), 70.2 (CH_2), 70.0 (CH_2), 69.7 ($ArOCH_2\underline{C}H_2$), 67.8 ($ArOCH_2$), 60.9 (CH_2OH), 19.3 (Ar-CH_3), 15.1 (Ar-CH_3). HRMS (ESI) *m/z*: $[M + Na]^+$ calcd for $C_{18}H_{30}O_6Na^+$ 365.1940, found 365.1925.

14-(2,4-Dimethyl-6-mercuryphenoxy)-3,6,9,12-tetraoxatetradecan-1-ol (1-Hg).

Compound **3** (65 mg, 0.19 mmol) and mercuric acetate (122 mg, 0.38 mmol) were allowed to react in CD_3OD (3.0 mL) at 50 °C overnight. Water (5.0 mL) was added and the resulting solution was filtered. The filtrate was fractioned by reverse phase chromatography on a Sunfire Hyperprep column (10 × 250 mm, 5 μm) using a gradient elution with a mixture of acetonitrile (30—50% over 30 min) and 50 mM

aqueous ammonium formate. Compound **1-Hg** was obtained in 56% yield (58 mg). Formation of a dimer containing a C-Hg-C cross-link during lyophilization of the collected fractions, presumably by the known symmetrization reaction[24], could not be completely avoided. ^{1}H NMR (500 MHz, D_2O) *δ*: 6.92 (s, 1H, Ar-H), 6.85 (s, 1H, Ar-H), 4.02 – 3.31 (m, 20H, 10 × CH_2), 2.18 (s, 3H, Ar-CH_3), 2.13 (Ar-CH_3). ^{13}C NMR (126 MHz, D_2O) *δ*: 159.6 (Ar-C1), 135.8 & 131.9 (Ar-C3 & Ar-C5), 134.8, 134.2 & 130.4 (Ar-C4 & Ar-C2 & Ar-C6), 72.8 (CH_2), 71.8 (CH_2), 69.9 (CH_2), 69.7 (CH_2), 69.7 (CH_2), 69.6 (CH_2), 69.6 (CH_2), 69.5 (CH_2), 69.5, 60.4 (CH_2OH), 20.3 (Ar-CH_3), 16.3 (Ar-CH_3). HRMS (ESI) *m/z*: $[M]^+$ calcd for $C_{18}H_{29}O_6Hg^+$ 543.1665, found 543.1693.

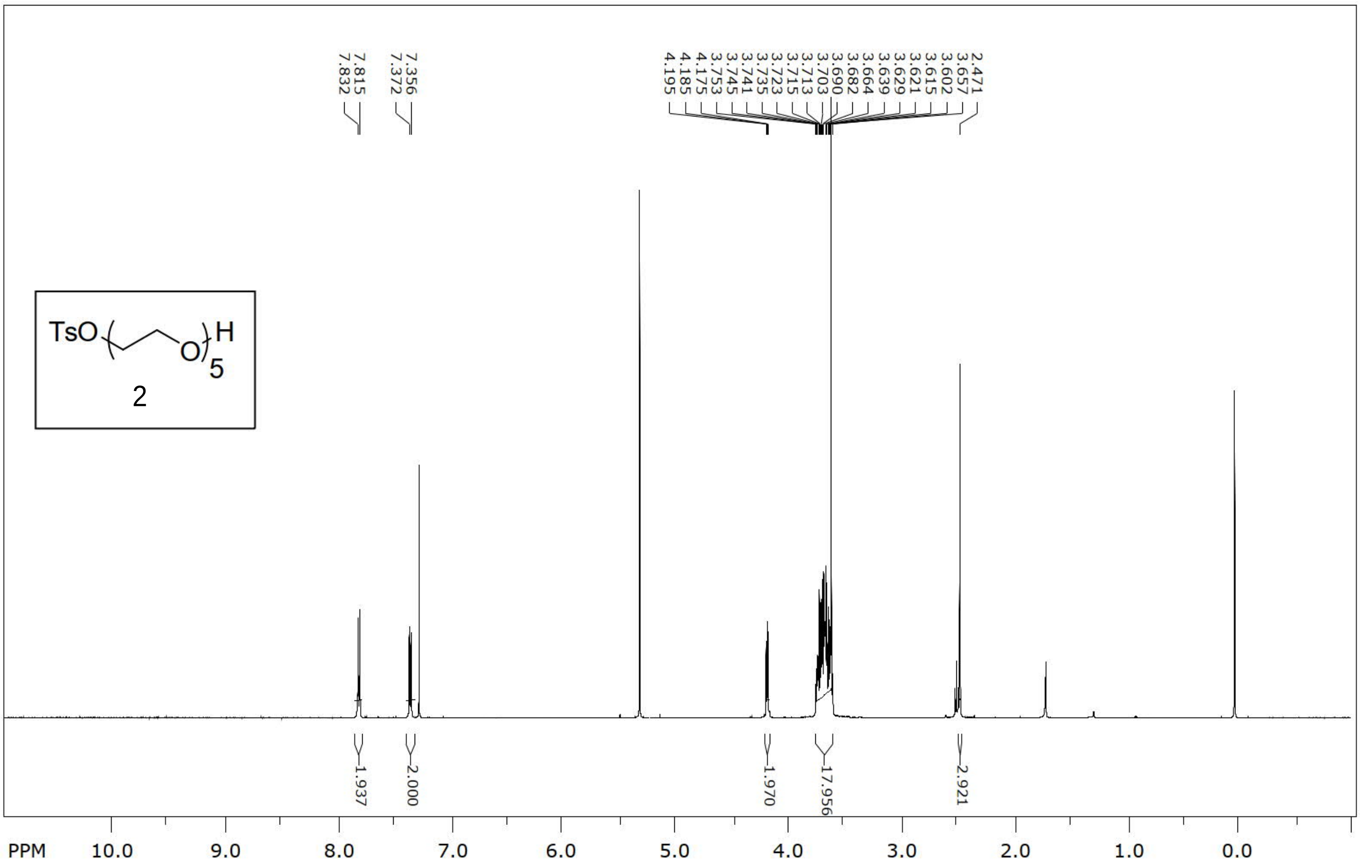

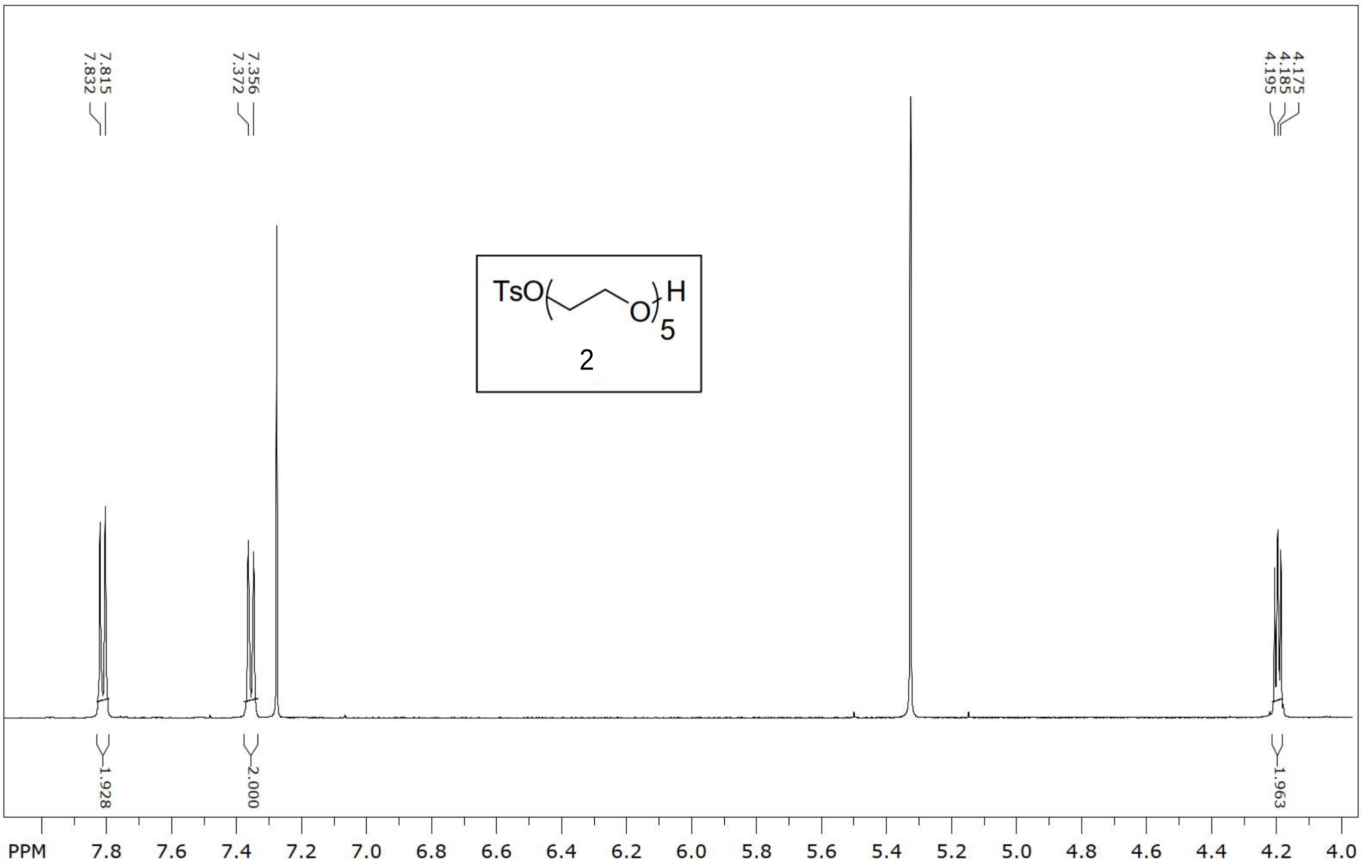

Figure S2. ^{1}H NMR spectra (500 MHz, $CDCl_3$) of compound **2**.

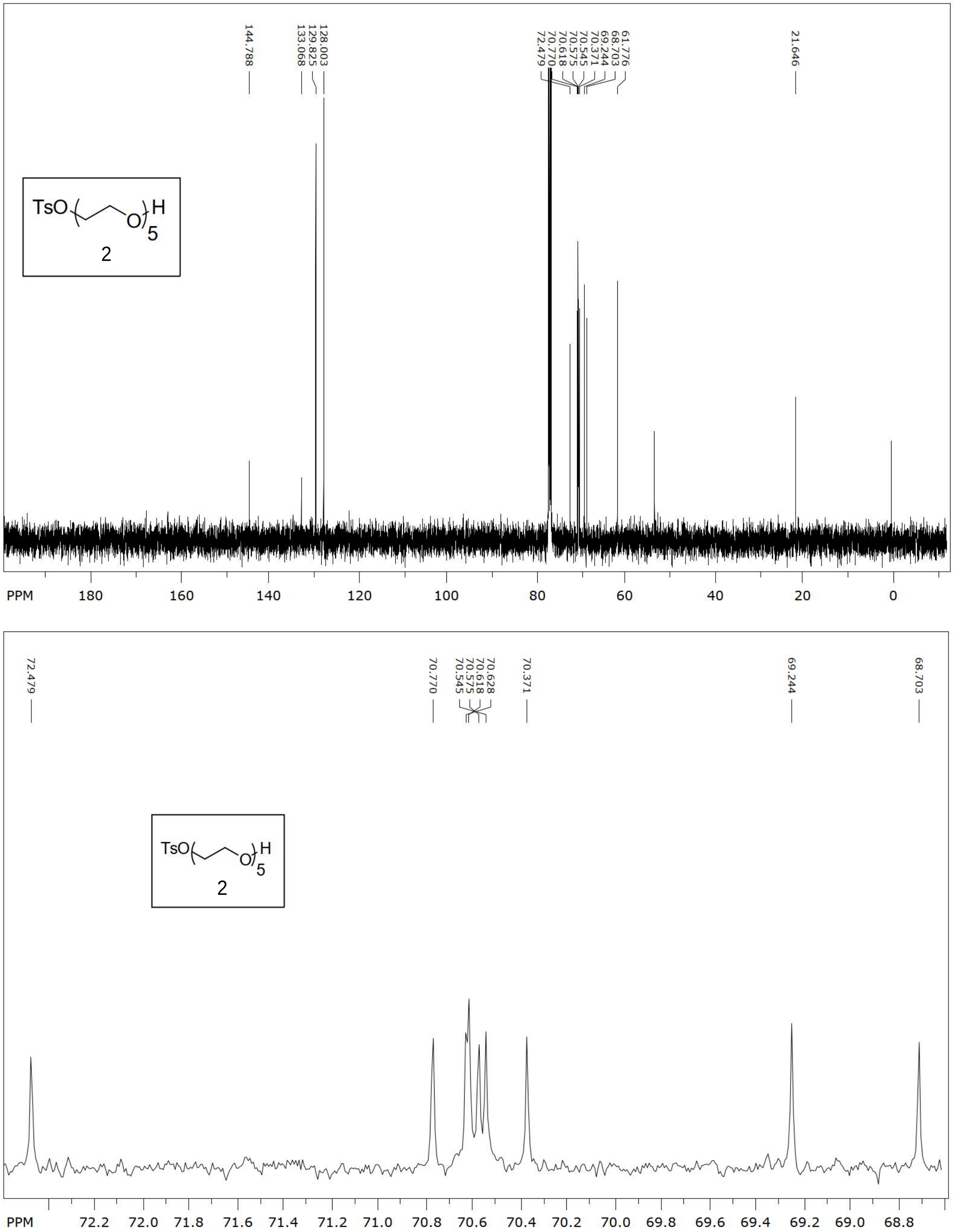

Figure S3. ^{13}C NMR spectra (126 MHz, CDCl3) of compound **2**.

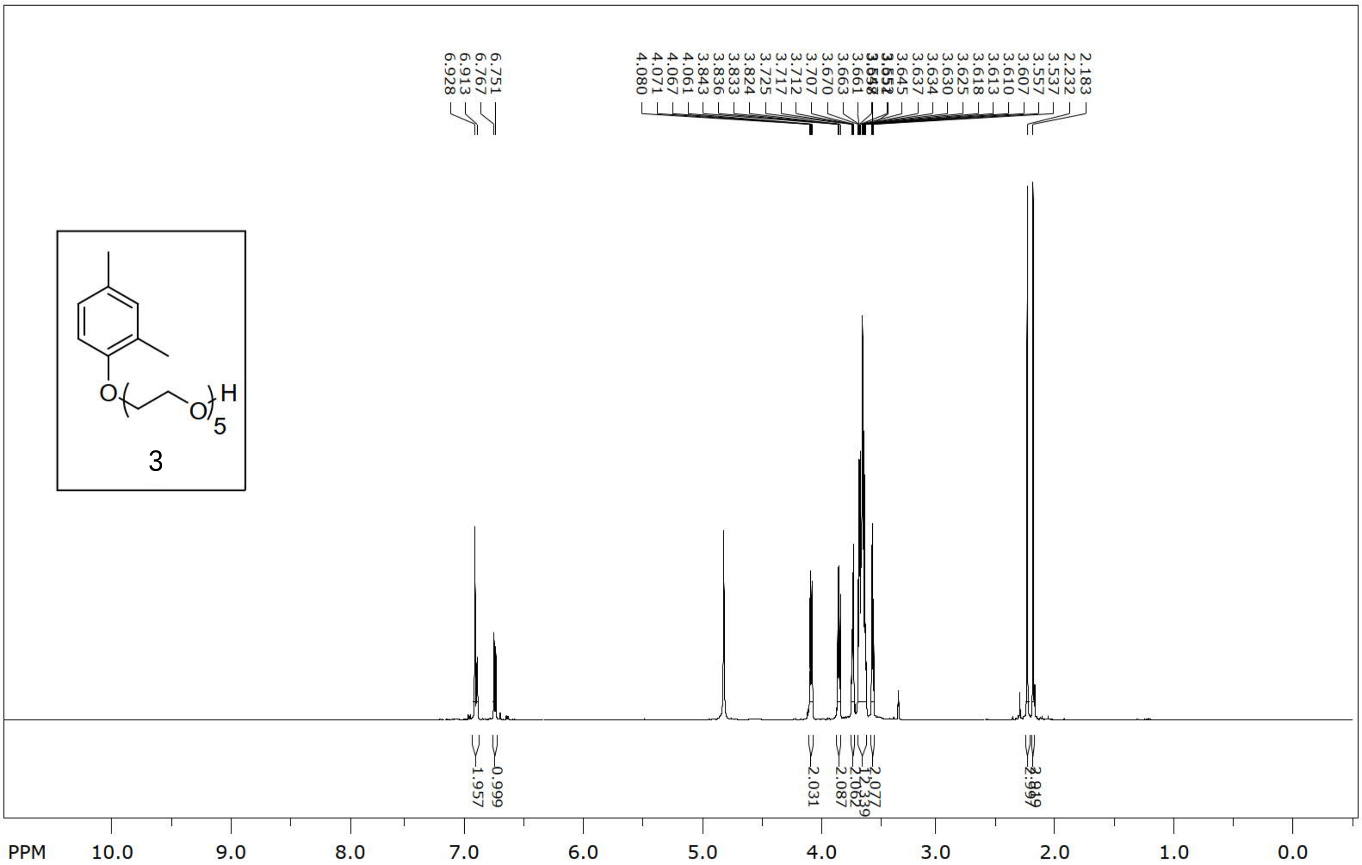
3
PPM 10.0 9.0 8.0 7.0 6.0 5.0 4.0 3.0 2.0 1.0 0.0
1.957
0.999
2.031
2.087
2.062
12.339
2.077

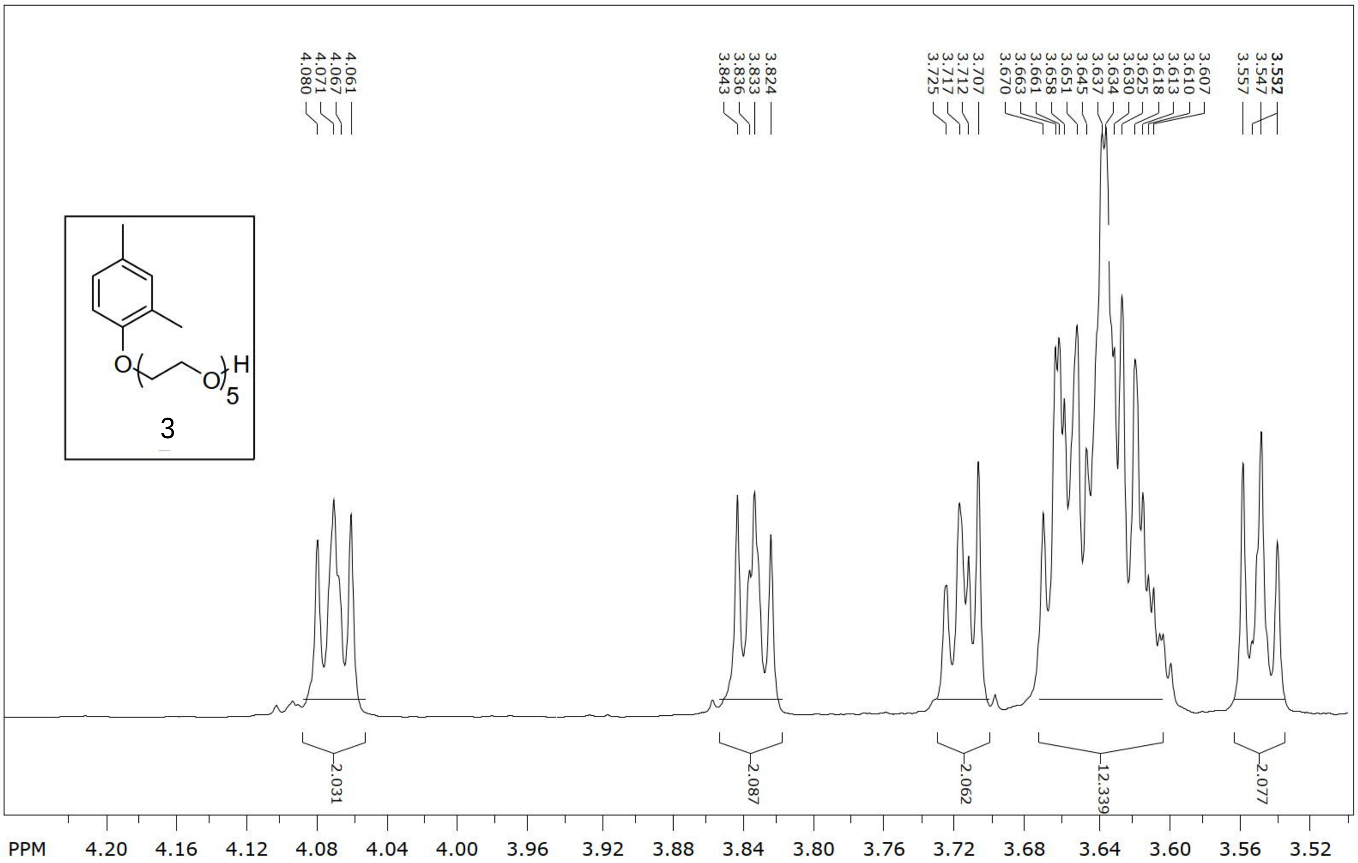
3
PPM 4.20 4.16 4.12 4.08 4.04 4.00 3.96 3.92 3.88 3.84 3.80 3.76 3.72 3.68 3.64 3.60 3.56 3.52
2.031
2.087
2.062
12.339
2.077

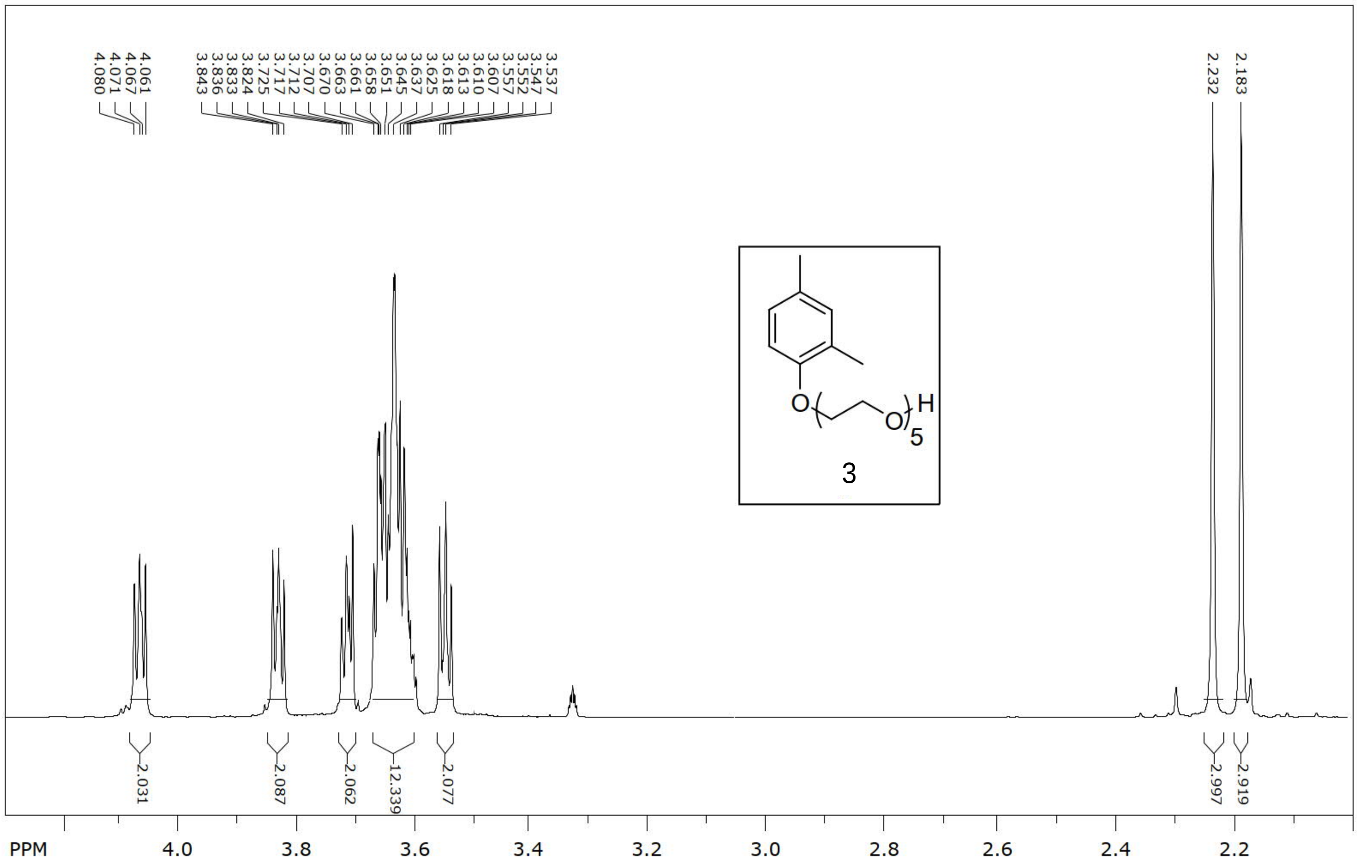

Figure S4. ^{1}H NMR spectra (500 MHz, CD_3OD) of compound **3**.

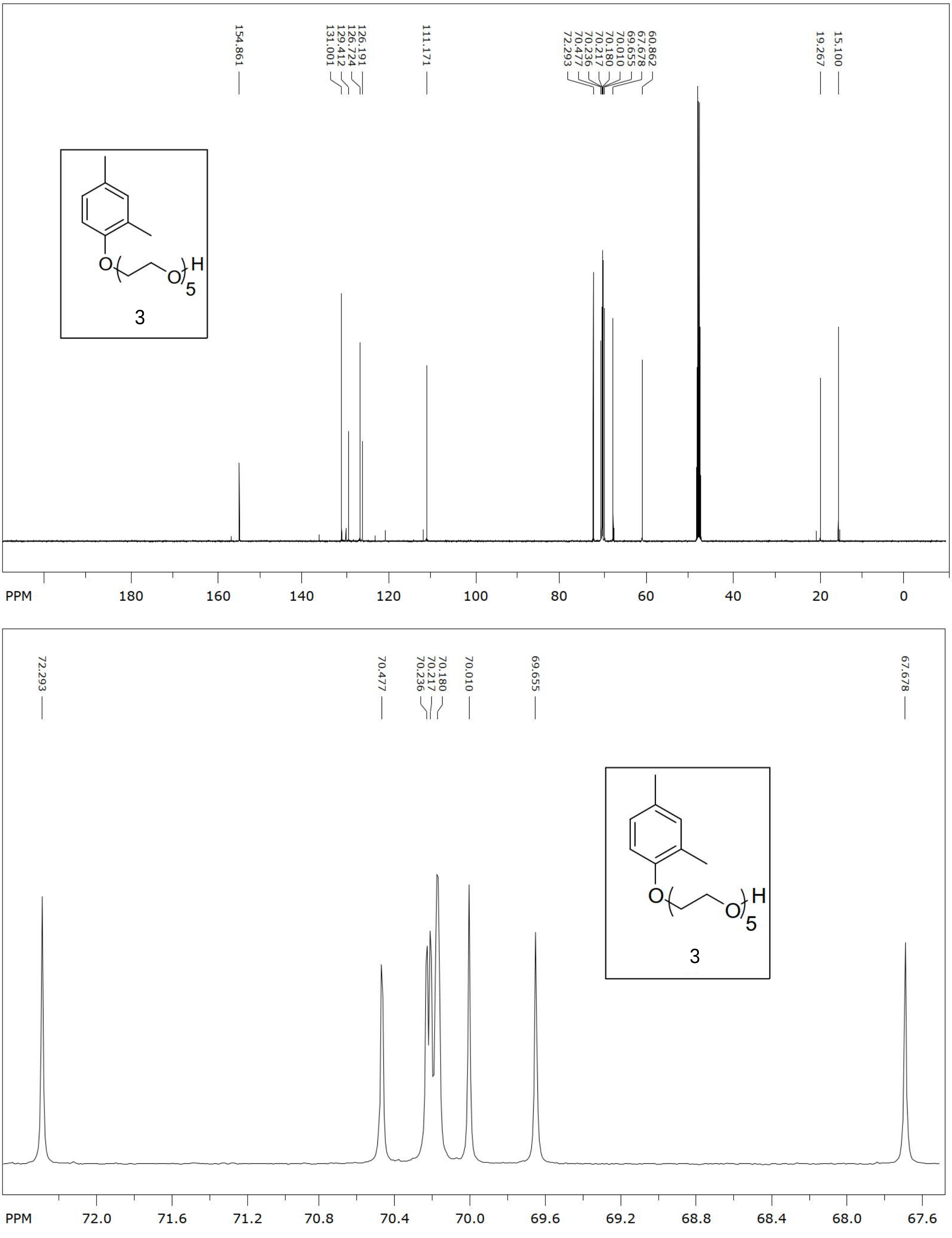

Figure S5. ^{13}C NMR spectra (126 MHz, CD_3OD) of compound **3**.

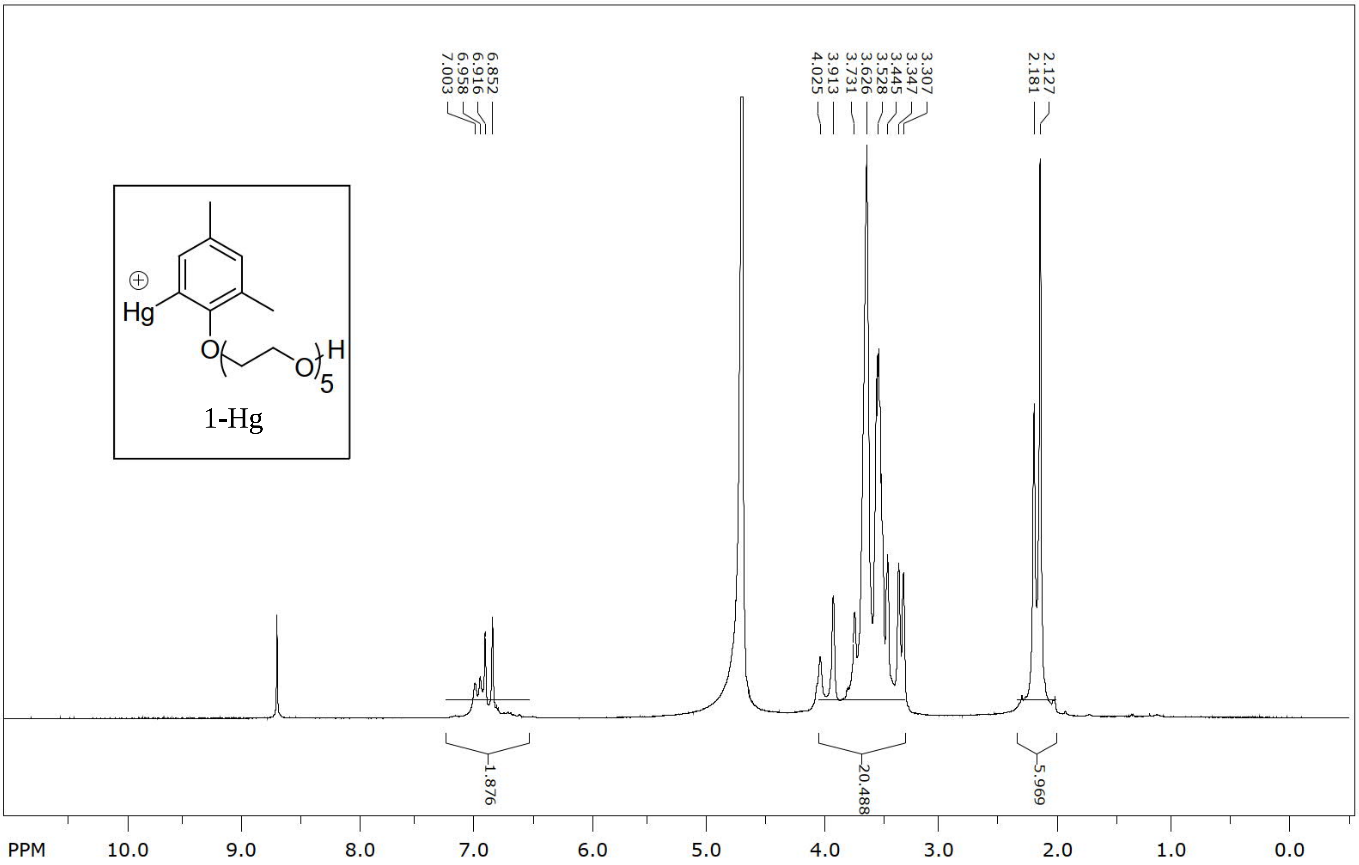

Figure S6. ^{1}H NMR spectrum (500 MHz, D_2O) of compound **1-Hg**.

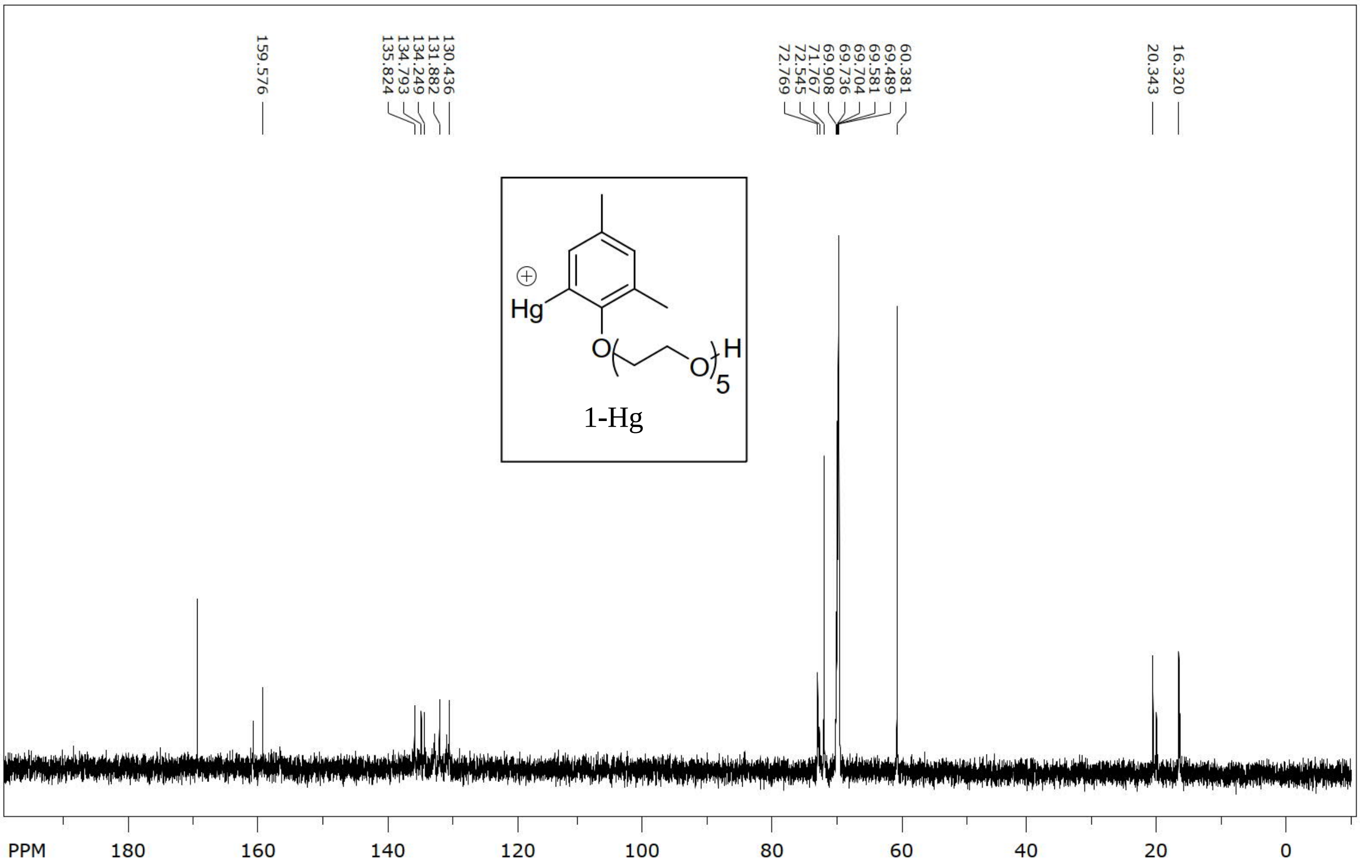
159.576
135.824
134.793
134.249
131.882
130.436
72.769
72.545
71.767
69.908
69.736
69.704
69.581
69.489
60.381
20.343
16.320
Hg
O
H
5
1-Hg
PPM
180
160
140
120
100
80
60
40
20
0

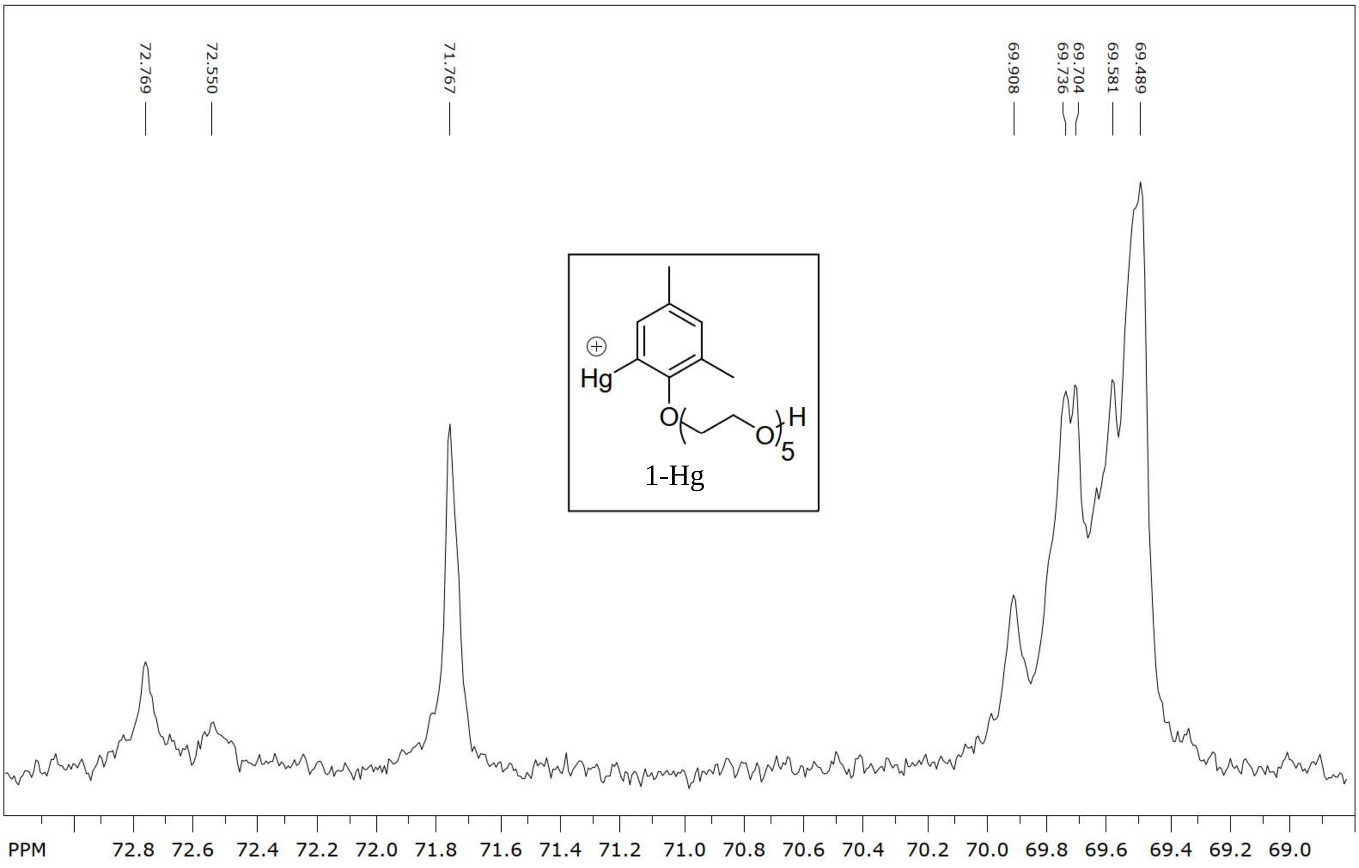
72.769
72.550
71.767
69.908
69.736
69.704
69.581
69.489
Hg
O
H
5
1-Hg
PPM
72.8 72.6 72.4 72.2 72.0 71.8 71.6 71.4 71.2 71.0 70.8 70.6 70.4 70.2 70.0 69.8 69.6 69.4 69.2 69.0

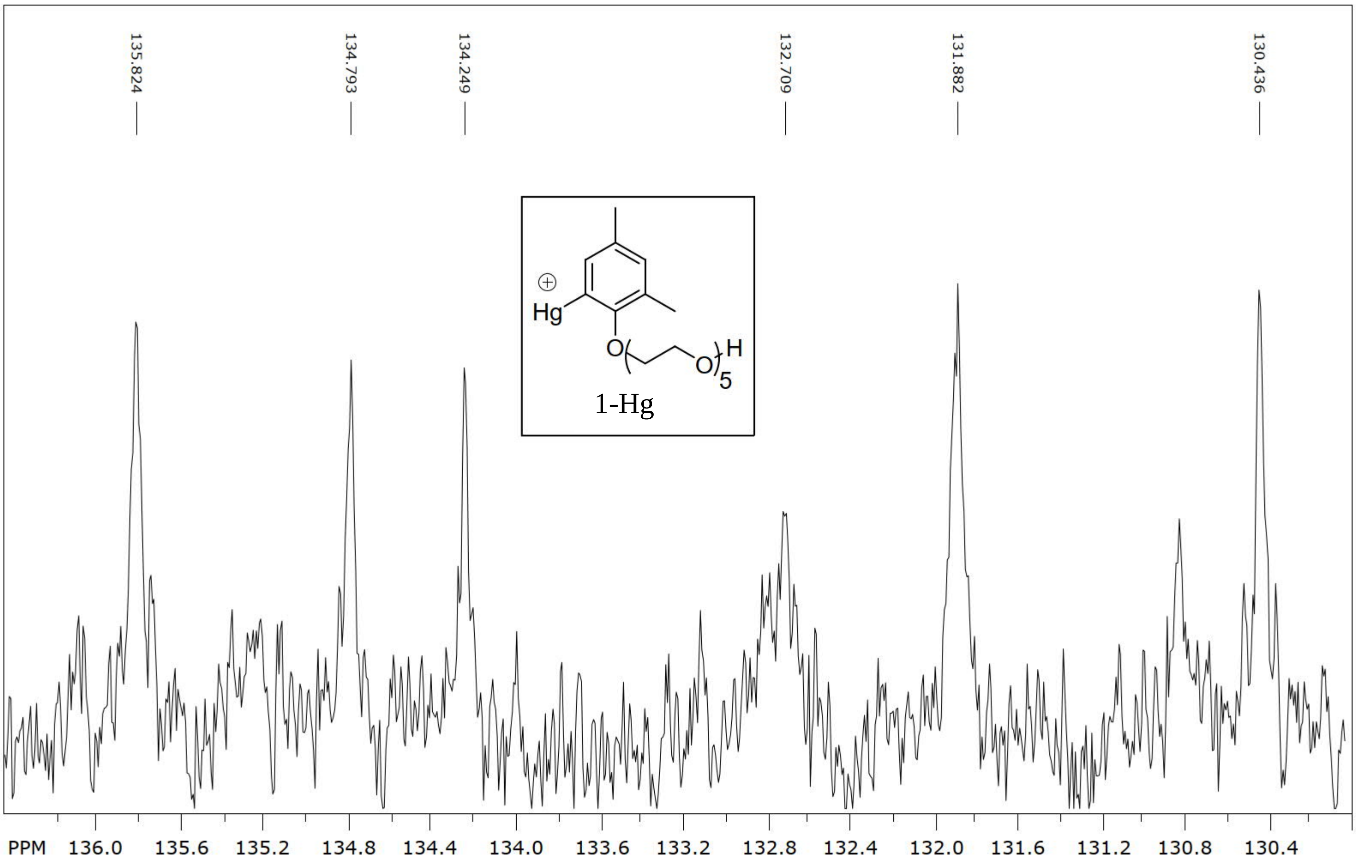

Figure S7. ^{13}C NMR spectra (126 MHz, D_2O) of compound **1-Hg**.

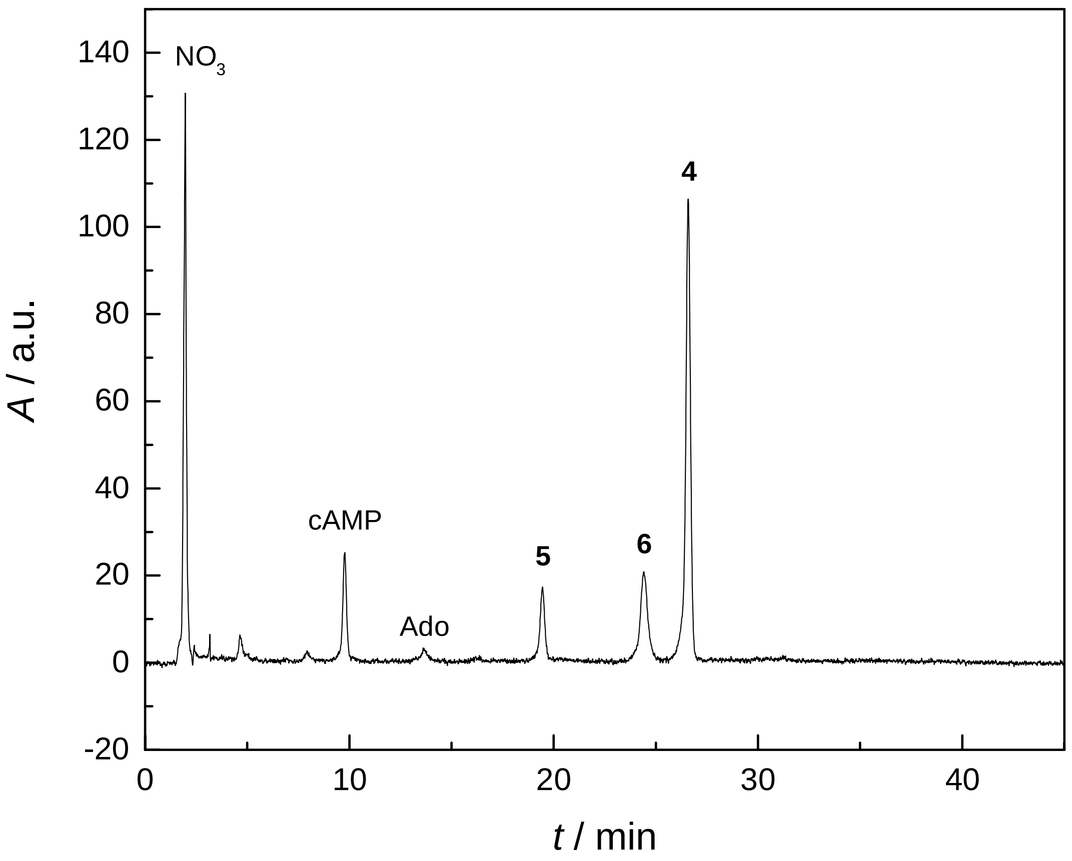

Figure S8. HPLC trace of a product mixture of cleavage and isomerization of **4** and **5**; Thermo Scientific Aquasil column (4 × 150 mm, 5 µm); linear gradient of acetonitrile (3—15% over 15 min) in 50 mM aqueous ammonium acetate; flow rate = 1.0 mL min^{-1}; λ = 260 nm.
